# Practical early cancer detection: distinguishing stable from unstable genomes in pre-cancerous tissues

**DOI:** 10.1038/s41416-020-01142-7

**Published:** 2020-11-06

**Authors:** Sarah Killcoyne, Rebecca C. Fitzgerald

**Affiliations:** 1 Medical Research Council Cancer Unit, Hutchison/Medical Research Council Research Centre, University of Cambridge, Cambridge, UK; 2 European Molecular Biology Laboratory, European Bioinformatics Institute (EMBL-EBI), Hinxton, UK

**Keywords:** Diagnostic markers, Cancer genomics

## Abstract

Barrett’s oesophagus has been known for many years to display early changes to the genome consistent with the risk for oesophageal adenocarcinoma. Recently we have shown that this information can be used without knowledge of individual gene mutations to accurately predict a patient’s future risk of malignant progression.

## Main

Early detection of cancer offers individual patients the best chance of long-term survival, and improved quality of life since treatment is usually less invasive. This is the driving force behind screening initiatives, and researchers’ continual efforts to refine our understanding of patient risk. Large genomic sequencing efforts in the last decade have provided molecular evidence that carcinogenesis occurs over many years,^1^ and that genetic mutations predisposing a patient to cancer may be found in physiologically normal tissues.^2,3^ This has offered positive evidence that genomics can offer a route to early detection of cancers. However, early detection must be balanced with overdiagnosis in pre-cancerous tissues that are unlikely to ever become malignant.

One of the difficulties in implementing early detection screening based on molecular data is the accessibility of pre-cancerous tissues, especially those where longitudinal samples could provide time-specific information showing transformation or stability. Lesions that are easy to access, such as skin, may also be easier to treat than observe, while tissues that require highly invasive clinical sampling methods (i.e. prostate needle biopsies) are going to be more difficult tissues from which to generate longitudinal data. Current methods for surveillance of the oesophagus involves sampling easily identifiable pre-malignant tissue over long periods of time. In the oesophageal premalignancy, called Barrett’s oesophagus, surveillance and early detection has focused on clinical risk stratification (i.e. patient age, length of the Barrett’s segment) and histopathology of the tissue.^4^ Considering the low annual risk of histopathological transformation, it is likely that many patients are undergoing invasive procedures unnecessarily.

Improving our understanding of patient risk in the context of oesophageal cancer has led to a number of efforts to identify sensitive biomarkers. Early genomics work^5^ showed that the genomes of Barrett’s tissues could display significant copy number changes, and subsequent studies confirmed aneuploidy and cellular clonality^6,7^ as features of malignant transformation. These led to attempts at patient stratification using immunohistochemistry biomarkers, measures of aneuploidy, panels of gene mutations,^8^ or methylation markers.

Our recent study^9^ has taken what has been learned about the genomics of Barrett’s in recent decades including the high rate of aneuploidy, increased tissue clonality, and low recurrence of mutations between patients, to generate a genome-wide statistical approach to risk stratification. This was enabled through the use of low-cost sequencing technologies ($200 per sample) directly on the patient samples that were already being collected and stored. Instead of selecting a single feature to stratify our patients, we used a method of regression that derived the features relating to malignant transformation from the data directly. This means rather than use explicit biomarkers, such as direct measures of aneuploidy, loss-of-heterozygosity, or clonality, the effects of these processes were modelled from the patient genome. In effect, it generated a measure of mutational complexity that sensitively predicted patient risk without requiring individual pre-specified biomarkers to be represented in a sample. More importantly, by classifying the relative risks from the model, we found that 50% of high-risk patients could be identified ≥8 years before malignant transformation. The closer we got to the patient developing cancer, the more accurately the model performed, with 78% identified 2 years prior, and 85% a year before their diagnosis. Equally, patients with stable genomes could be classified as low risk within the first 2 years of surveillance, potentially decreasing the rate of invasive procedures by more than 50%.

**Fig. 1:**
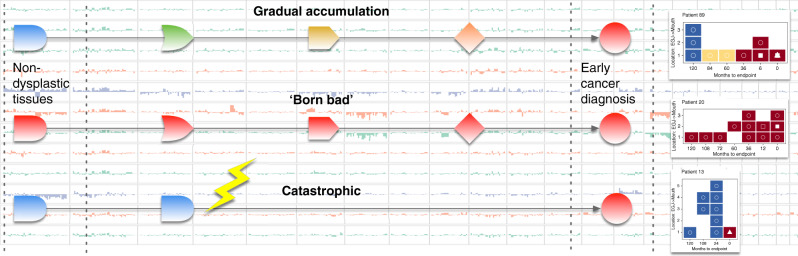
Patterns of progression. Our study suggests that there may be three different genomic patterns of progression: the gradual accumulation of mutations and copy number rearrangements, increasing a patient’s risk over time; tissues that display high rates of genomic instability from the earliest samples and throughout surveillance suggesting these tissues are ‘born bad’ or primed to progress; and evidence that in some patients a catastrophic event may lead to early cancer after a period of genomic stability.

These findings were surprising. The risks appeared to be independent of the histopathology of the samples, and highly consistent over many years. Several patterns of progression appeared to emerge as well: many patients were high risk from their earliest samples indicating that their Barrett’s was primed to be malignant (i.e. ‘born bad’); some patients showed evidence of gradual genomic disorder leading to cancer; and a small number of patients appeared stable until their final sample, suggesting a catastrophic genomic change (Fig. 1). Ensuring that this risk model is accurate across these different patterns of progression will necessarily involve analysing more patients and evaluating how these risks relate to the clinical factors that are currently in use. In this study we used a nested case−control design, which means that we matched progressors and non-progressors based on their demographic and clinical factors such as the length of the Barrett’s segment. In future it would be interesting to see the extent to which clinical factors could add to the model. Additional information from the morphological features obtained by histopathology could also be included in the model. Merging these different types of information, always bearing in mind the clinical feasibility of the approach, will provide more accurate risk stratification for clinicians and patients to guide decision making.

Barrett’s provides a unique opportunity to analyse a pre-malignant tissue as it transforms. It may also enable researchers to look for similar signatures in other cancer types. Our current understanding of the evolution of most cancers suggests that very early events often include chromosomal instability. In the case of some tumours these may be highly specific, as in glioblastoma where chromosomes 7, 19, or 20 show evidence of early chromosomal gains. While normal tissues without known malignancies may show a patchwork of cell populations with detectable mutational and copy number differences.^3^ As these types of studies mature and more data across normal and cancer tissues becomes available, the type of risk classification performed on Barrett’s tissues will likely be possible for many other cancers.

Completion of the Human Genome Project in 2001 brought great hopes for an era of medicine informed by an understanding of genomics. Through the International Cancer Genome Consortium many large sequencing projects since then have been carried out with the hope of genomics-led diagnostics and therapeutics.^10^ All of these have advanced our understanding of the genomic complexity of cancer; however, for the most part this has helped only a small subset of patients with specific mutations. Our recent study offers a practical example of how genomic medicine can be implemented for early detection.

## Data Availability

Not applicable.
